# Histone Deacetylase 3-Mediated Inhibition of microRNA-19a-3p Facilitates the Development of Rheumatoid Arthritis-Associated Interstitial Lung Disease

**DOI:** 10.3389/fphys.2020.549656

**Published:** 2020-12-04

**Authors:** Hui Yuan, Li Jiao, Nan Yu, Haifeng Duan, Yong Yu, Yanrong Bai

**Affiliations:** ^1^Department of Rheumatic Nephropathy, Affiliated Hospital of Shaanxi University of Chinese Medicine, Xianyang, China; ^2^Yanching Institute of Technology, Langfang, China; ^3^Shaanxi University of Chinese Medicine, Xianyang, China; ^4^Department of Imaging, Affiliated Hospital of Shaanxi University of Chinese Medicine, Xianyang, China

**Keywords:** rheumatoid arthritis-associated interstitial lung disease fibrosis, microRNA-19a-3p, histone deacetylase 3, interleukin 17 receptor, lung fibroblast

## Abstract

Histone deacetylase (HDAC) has been implicated in rheumatoid arthritis (RA) progression. We investigated the roles of histone deacetylase 3 (HDAC3) involved in RA-associated interstitial lung disease (ILD) fibrosis. Firstly, we measured the expression of HDAC3 and interleukin 17 receptor A (IL17RA) in lung tissue samples from normal controls, idiopathic pulmonary fibrosis (IPF) patients, and RA-ILD patients. Next, chromatin immunoprecipitation (ChIP) and dual luciferase reporter assay were employed to detect the interaction between HDAC3 and microRNA-19a-3p (miR-19a-3p) and between miR-19a-3p and IL17RA. Further, immunohistochemistry was used to localize HDAC3 and IL17RA expression in lung tissues. Additionally, functional assays were conducted followed by expression determination of HDAC3, miR-19a-3p, and IL17RA with reverse transcription quantitative PCR (RT-qPCR) and Western blot analysis. The effect of HDAC3 on RA-ILD in the constructed RA-ILD mouse model was also studied based on arthritis assessment. We found overexpressed HDAC3 and IL17RA as well as silenced miR-19a-3p in RA-ILD mouse model and RA-ILD patients. In the mouse model, HDAC3 downregulated miR-19a-3p in lung fibroblasts to promote the progression of RA-ILD fibrosis. In lung fibroblasts of RA-ILD mice, IL17RA was a target gene of miR-19a-3p. miR-19a-3p negatively regulated IL17RA, thereby increasing the expression of fibrosis markers, COL1A1, COL3A1, and FN, in lung fibroblasts of mice. Taken together, HDAC3 upregulated IL17RA expression by targeting miR-19a-3p to facilitate the RA-ILD fibrosis development, which sheds light on a new HDAC3/miR-19a-3p/IL17RA axis functioning in RA-ILD fibrosis.

## Introduction

Rheumatoid arthritis (RA) is a systemic and chronic disease featured by inflammation of synovium, erosions of bones, pannus formation, and destruction of joints (Wells et al., 2019) in human. It is a chronic inflammatory rheumatic disease characterized by modification of lipids profile and an elevated risk of inflammation-related cardiovascular events (Audo et al., 2018). Even though advances have been achieved recently in treating this disease, there still exists a large demand for alternative treatments (Rana et al., 2018). The detection results of high-resolution computed tomography (HRCT) show that interstitial lung disease (ILD) occurs in more than 60% of RA patients, and ILD is a primary cause of death in RA patients (Juge et al., 2017). Idiopathic pulmonary fibrosis (IPF) is one kind of chronic and irreversible ILD, which has been with poor prognosis and few therapies (Vasarmidi et al., 2018). ILD happens in many patients with autoimmune myositis, but the factors of susceptibility remain unknown (Johnson et al., 2017). Advanced fibrosing ILD is invasive and is associated with heavy burden of symptoms and poor prognosis (Ahmadi et al., 2016). Early diagnosis of ILD is critical for effectively treating RA-associated ILD (RA-ILD) in clinical profiles (Yu et al., 2019).

Histone deacetylase (HDAC) is a kind of enzymes regulating gene expression through removing the acetyl group of histones (Jang et al., 2018). HDACs deacetylate various non-histone proteins such as transcription factors, which were involved in controlling the growth, differentiation, and apoptosis of cells (Ganai, 2018). HDACs may play important roles in RA progression. For example, HDAC1 was upregulated in the synovium of patients with RA (Li et al., 2018). Meanwhile, studies have shown that HDAC3 negatively regulates the expression of microRNA (miRNA) cluster miR-17-92 through deacetylation of promoter regions (Wang et al., 2016). miRNAs are small non-coding RNAs (Di Leva et al., 2014). Recently, miRNAs have been implicated as promising bio-markers for early diagnosis of diseases (Ntoumou et al., 2017). Abnormal expression of multiple miRNAs has been found in cells, tissues, and biological fluids in RA patients (Evangelatos et al., 2019). For example, miR-19a-3 is reported lowly expressed in both RA and specific pulmonary fibrosis (Chen et al., 2016, 2019). It is reported that miRNAs show the ability of regulating target gene translation through binding to the 3'-untranslated region (UTR) of miRNAs and play a critical role in biological functions (Evangelatos et al., 2019). Interleukin (IL) 17 family cytokines are a type of important regulators in response to mucosal immune (Wang et al., 2014). Importantly, IL-17 receptor A (IL17RA) is reported to play a direct role in lung fibrosis that may be particularly implicated in RA-ILD (Zhang et al., 2019). Besides, miR-155 can lead to the occurrence of RA through modulating the production of chemokines and stimulating the expression of pro-inflammatory chemokines receptor (Elmesmari et al., 2016).

Taking all the published findings into consideration, it was hypothesized that HDAC3, miR-19a-3p, and IL17RA are possible immune response regulators in the development of RA-ILD fibrosis. Therefore, our study was designed and conducted to explore the mechanism of HDAC3, miR-19a-3p, and IL17RA in RA-ILD fibrosis.

## Materials and Methods

### Ethics Statement

This study was performed with the approval of the Ethics Committee of Affiliated Hospital of Shaanxi University of Chinese Medicine. All patients signed informed consent with the approval of the Review Committee. The experimental procedure and animal use program were approved by the Animal Ethics Committee.

### Patient Enrollment

A total of 16 patients with specific pulmonary fibrosis (IPF group) and 28 patients with RA-ILD (RA-ILD group) diagnosed and hospitalized in the Department of Respiratory Medicine of Affiliated Hospital of Shaanxi University of Chinese Medicine from January 2017 to 2019 were included, and 20 patients who underwent fiberoptic bronchoscopy for unexplained hemoptysis in Affiliated Hospital of Shaanxi University of Chinese Medicine during the same period were selected as the control group. Lung tissue samples were obtained by fiberoptic bronchoscopy.

IPF diagnosis was based on the American Thoracic Society/European Respiratory Society clinical and HRCT criteria. According to the above criteria, patients with any known cause of pulmonary fibrosis, such as systemic connective tissue disease, were excluded by immunological screening and clinical rheumatological evaluation. All IPF patients were newly diagnosed and had not received any treatments before. The criteria for RA-ILD diagnosis were based on the American College of Rheumatology RA classification revision standard in 1987 (Samara et al., 2017). The HRCT findings of RA-ILD patients showed definitive pulmonary interstitial lesions, and the diagnosis of RA-ILD patients was performed by two independent evaluators. The control patients (had smoking history) had no significant pulmonary complications, and the results of their bronchoscopy and cytology were normal.

### Mouse Model of RA-ILD

A total of 30 SKG ZAP-70W163C – mutant BALB/c mice (age: 5–9 weeks and weight: 16–24 g) were selected, and 10 of them were injected with physiological saline as controls, and 20 of them were subjected to model establishment. The experimental mice were injected intraperitoneally with 2 mg of zymosan (Sigma-Aldrich, St. Louis, MO, United States) to induce RA-ILD (Redente et al., 2018), followed by the evaluation of arthritic diseases and lung diseases. Fifteen mouse models of RA-ILD were successfully constructed. A total of 10 RA-ILD mice were injected with 10 μl of 3.5 × 10^9^ pyrococcus furiosus adenoviral vector (Sangon Biotech, Shanghai, China) *via* tail vein to transfect si-HDAC3 for RA and ILD study.

### Cell Culture and Grouping

The lung fibroblasts of RA-ILD mice were isolated and cultured by enzyme treatment. The cells were cultured in DMEM containing 10% fetal bovine serum (FBS) at 37°C with 5% CO_2_ for 48 h. The 16S rRNA coding region in the highly conserved mycoplasma genome was amplified by PCR using designed specific primers. Primary mouse lung fibroblasts were confirmed to be free of mycoplasma contamination and passaged for 3–5 times for following experiments.

Next, 1 × 10^5^ or 4 × 10^5^ mouse lung fibroblasts were seeded in 24‐ or 6-well plates, respectively. After growing to 30–50% confluence, the cells were transfected with NC mimic, miR-19a-3p mimic, si-NC, si-HDAC3, or si-IL17RA (all designed and synthesized by Guangzhou RiboBio Co., Ltd., Guangzhou, China) according to the lipofectamine 2000 instructions (11668-019, Invitrogen, New York, CA, United States). At 12 h after transfection, the cells were cultured at 48 h at 37°C in 5% CO_2_ for RNA extraction.

### Hematoxylin-Eosin and Masson’s Staining

Lung tissue samples of mice in each group were washed with normal saline, and fixed with 4% paraformaldehyde for 30–50 min, followed by washes, dehydration, clearance, waxing, embedding, and sectioning. Tissue sections were attached to slide, baked in a 45°C incubator, dewaxed, and then washed with gradient alcohol and distilled water followed by 5 min staining with hematoxylin solution. Afterward, the sections were washed with water for 3 s, differentiated by 1% hydrochloric acid ethanol for 3 s, and stained with 5% eosin solution. Then, the sections were dehydrated, cleared, and sealed.

For Masson’s staining, the sections were stained with picric acid-sirius red at room temperature for 30 min and inhibited by hematoxylin (CAS no. PT003; Shanghai Bogoo Biotechnology Co., Ltd., Shanghai, China) at room temperature for 2 min. Finally, the sections were observed by a microscope (XPT-480; Shanghai Zhongheng, co., Ltd., Shanghai, China) and analyzed by Image Pro 6 (Media cybernetics Inc., Bethesda, MD, United States).

### Cell Immunofluorescence

Slides of primary lung fibroblasts were prepared and fixed with 40 g/L paraformaldehyde at room temperature for 15 min, washed three times with PBS, and blocked with 10% goat serum. The sections were incubated with primary anti-rabbit antibodies against α-SMA (ab5831, 1:500, Abcam, UK), HDAC3 (ab7030, 1:500, Abcam, UK), and IL17RA (ab180904, 1:500, Abcam, UK) overnight at 4°C. Then, the sections were incubated with secondary antibodies against Alexa Fluor® 647-labeled fluorescence goat-anti-rabbit antibody against immunoglobin G (IgG) H and L (ab150079, 1:1,000, Abcam, Cambridge, UK) and FITC-labeled fluorescence goat-anti-mouse antibody against IgG H and L (ab6785, 1:1,000, Abcam, Cambridge, UK) for 45 min. Following three PBS washes, the sections were sealed with anti-fluorescent quenching mounting medium Vectashield (Vector Laboratories Inc., Burlingame, CA), and observed and photographed under a fluorescence microscope.

### Immunohistochemistry

Human lung tissues from controls, patients with IPF, and patients with RA-ILD were fixed with 4% paraformaldehyde, mounted to conventional paraffin embedding, sliced serially as 4 μM sections, and dewaxed. After antigen retrieval, normal goat serum was added for blocking. The lung tissues of control patients were used as negative controls during the analysis, and stained with Histostain™ SP-9000 immunohistochemical staining kit (Zymed Laboratories, San Francisco, CA, United States). The tissues were added with primary antibody rabbit anti HDAC3 (ab7030, 1:500, Abcam) and rabbit anti IL17RA (ab180904, 1:500, Abcam). After incubating with secondary antibody (ab9482, 1:5,000, Abcam) at 37°C for 30 min, horseradish-labeled working fluid was added to the tissues, and the tissues were developed by diaminobenzidine. The tissues were counterstained with hematoxylin for 1 min, and photos were taken after mounting. Five representative high power fields (positive optical microscope, NIKON, Tokyo, Japan) were selected for counting the cells with brown or yellow cytoplasm to calculate the positive rate.

### Dual Luciferase Reporter Assay

The wide type (WT) target site sequence of the 3'-UTR region of IL17RA and mutation (MUT) after site-directed mutagenesis of the WT target site were synthesized. The psiCHECK™-2 plasmid (Promega, Madison, WI, United States) was digested by restriction endonucleases, and the artificially synthesized target gene fragments WT and MUT were inserted into the psiCHECK™-2 vector, respectively. The empty plasmids were transfected as negative controls, and the sequence proved luciferase reporter plasmids WT and MUT were co-transformed into 293 T cells with mimic NC or miR-19a-3p mimic, respectively. After 48 h of transfection, the cells were collected and lysed. The supernatant was also collected. Relative light units were measured using a luciferase assay kit (KA3784, Abnova, Taipei, Taiwan, China).

### Reverse Transcription Quantitative PCR

The groups of mouse lung fibroblasts and lung tissue samples were lysed using the TRIzol kit (Invitrogen, ThemoFisher, Carlsbad, CA, United States) to extract total RNA. Reverse transcription of total RNA was performed using PrimeScript RT reagent kit (Takara, Dalian, China). A fluorescent quantitative PCR operation was carried out using cDNA as a template and referring to the instruction of SYBR® Premix Ex Taq™ II (Tli RNaseH Plus) kit (Takara Holdings Inc., Kyoto, Japan). The primers were synthesized by Guangzhou RiboBio Co., Ltd., (Guangzhou, China). The specific sequences are shown in Table 1. Glyceraldehyde-3-phosphate dehydrogenase (GAPDH) was used as an internal reference gene. The 2^(−ΔΔCT) indicates expression of the interested gene, ΔΔCT = CT (target) − CT (ref).

**Table 1:** Primer sequences for RT-qPCR.

Gene	Primer sequences (5'-3')
HDAC3 (humo)	F: 5'-TTGAGTTCTGCTCGCGTTACA-3'
	R: 5'-CCCAGTTAATGGCAATATCACAGAT-3'
HDAC3 (mmu)	F: 5'-TGATGACCAGAGTTACAAGCAG-3'
	R: 5'-GGGCAACATTTCGGACAG-3'
IL17RA (mmu)	F: 5'-AATACCACAGTTCCCAAGCCAG-3'
	R: 5'-CAGGTCTGCTACGGGCAAG-3'
miR-19a-3p (humo)	F: 5'-GGGGGGGTGTGCAAATCT-3'
	R: 5'-GTGCGTGTCGTGGAGTCG-3'
miR-19a-3p (mmu)	F: 5'-CAATCCTCTCAGGCTCAGTCC-3'
	R: 5'-TATGCTTGTTCTCGTCTCTGTGTC-3'
COL1A1 (mmu)	F: 5'-GCCAAGAAGACATCCCTGAAG-3'
	R: 5'-TGTGGCAGATACAGATCAAGC-3'
COL3A1 (mmu)	F: 5'-GCCAAGAAGACATCCCTGAAG-3'
	R: 5'-TGGACTGCTGTGCCAAAATA-3'
FN (mmu)	F: 5'-ACAGAAATGACCATTGAAGG-3'
	R: 5'-TGCAAGGCAACCACACTGAC-3'
U6 (mmu)	F: 5'-TCCGACGCCGCCATCTCTA-3'
	R: 5'-TATCGCACATTAAGCCTCTA-3'
U6 (humo)	F: 5'-GCTTCGGCAGCACATATACTAAAAT-3'
	R: 5'-CGCTTCACGAATTTGCGTGTCAT-3'
GAPDH (mmu)	F: 5'-TCCGCCCCTTCTGCCGATG-3'
	R: 5'-CACGGAAGGCCATGCCAGTGA-3'
GAPDH (humo)	F: 5'-GGGCTGCTTTTAACTCTGGT-3'
	R: 5'-TGGCAGGTTTTTCTAGCGG-3'

RT-qPCR, reverse transcription quantitative PCR; HDAC3, histone deacetylase 3; IL17RA, interleukin 17 receptor A; miR-19a-3p, micro-19a-3p; COL1A1, chain of type I procollage; COL3A1, chain of type III procollage; GAPDH, glyceraldehyde-3-phosphate dehydrogenase; F, forward; R, reverse.

### Western Blot

Human lung tissues from controls, patients with IPF, and patients with RA-ILD or mouse lung fibroblasts were lysed by lysis buffer (C0481, Sigma-Aldrich Chemical Company, St. Louis, MO, United States) to extract total protein. Protein loading buffer was added to the supernatant. After boiling for 5 min, 20 μg of protein sample was electroporated onto a polyvinylidene fluoride membrane by 10% sodium dodecyl sulfate polyacrylamide gel electrophoresis (Millipore, Billerica, MA, United States). The membrane was added with Tris-buffered saline containing Tween 20 (TBST) diluted primary antibodies IL17RA (ab180904, 1:1,000, Abcam), HDAC3 (ab219376, 1:1,000, Abcam), and GAPDH rabbit anti (ab181602, 1:10,000, Abcam) for incubation overnight at 4°C. HRP-labeled secondary antibody (ab99702, 1:1,000, Abcam) was added and incubated for 1 h. Enhanced chemiluminescence (Shanghai Baoman Biotechnology Co., Ltd., Shanghai, China) was used for developing. GAPDH was used as an internal reference. The Image J was used to analyze the luminosity of each band, and the ratio of the value of the target protein to the internal reference was calculated.

### Chromatin Immunoprecipitation

Mouse lung fibroblasts were cultured, and 1% formaldehyde was added when they reached 70–80% confluence. The cells were fixed for 10 min to cross-link the DNA to protein in the cells, and then randomly broken with ultrasonic treatment. The cells were sectioned and centrifuged at 13,000 rpm at 4°C. The collected supernatant was divided into two tubes. Negative control antibody rabbit anti-IgG (ab109489, 1:100, Abcam) and the protein-specific antibody mouse anti-HDAC3 (ab7030, 1:50, Abcam) were added, and the mixture was incubated overnight at 4°C. The endogenous DNA-protein complex was precipitated using Protein Agarose/Sepharose, and the supernatant was aspirated after brief centrifugation. The non-specific complex was washed off. The complex was decross-linked at 65°C overnight. The DNA fragments were purified by phenol/chloroform. The enrichment of miR-19a-3p promoter binding to HDAC3 was determined using the miR-19a-3p gene promoter fragment-specific primer. MiR-19a-3p promoter primer: forward: TTAGGCCTCGGGCCGC and reverse: AAACACAACTATGGAGAGACCCC.

### Statistical Analysis

The SPSS 21.0 version (IBM Corp., Armonk, NY, United States) was used for statistical analysis. The continuous data were expressed as mean ± SD. The difference between the two groups was analyzed by independent sample *t*-test. Comparisons among multiple groups were analyzed by one-way ANOVA followed by Tukey’s *post hoc* test. Comparison among groups at different time points was performed by repeated measures ANOVA, and Bonferroni was used for *post hoc* testing. Pearson’s correlation was used to analyze the correlation between HDAC3 and IL17RA expressions. The classified variables were shown in the number of cases using Fisher’s exact test. A value of *p* < 0.05 was statistically significant.

## Results

### High Expression of HDAC3 and IL17RA in Lung Tissues of Patients With RA-ILD

In order to investigate the pathogenesis of RA-ILD, we collected lung tissues of clinical patients for relevant detection. The main clinical presentation of the patients is shown in Table 2. Expressions of HDAC3 and IL17RA were examined in lung tissue samples from 20 controls, 16 patients with IPF, and 28 RA-ILD patients. Results of reverse transcription quantitative PCR (RT-qPCR) assay (Figures 1A,B) showed that HDAC3 and IL17RA expressions were much higher in RA-ILD patients than in controls. Further analysis (Figure 1C) revealed a positive correlation of expression between HDAC3 and IL17RA in RA-ILD. Western blot and immunohistochemistry (Figure 1D and Supplementary Figure 1A) showed that HDAC3 and IL17RA expression in lung tissue of RA-ILD patients was higher than that of controls. The above results supported that HDAC3 and IL17RA were both highly expressed in lung tissues of RA-ILD patients, and they might be involved in RA-ILD progression.

**Table 2:** Clinicopathological features of patients in the IPF, RA-ILD, and control groups.

	Control	IPF	RA-ILD	*p*
Number	20	16	28	
Age (years)	64.30 ± 8.81	57.00 ± 8.93	62.21 ± 9.70	^*^0.056; ^#^0.721
Gender(male/female)	11/9	10/6	16/12	^*^0.650; ^#^0.882
Non-smokers vs. ever-smokers	6/14	5/11	9/19	^*^0.935; ^#^0.875
Disease duration (years)		7.53 ± 4.77	7.02 ± 3.20	0.6735
FEV1%pred		68.39 ± 8.45	79.56 ± 17.28	0.02
FVC%pred		63.38 ± 16.65	77.48 ± 21.59	0.03
Dlco%pred		43.65 ± 8.94	58.38 ± 9.56	<0.001
ESR (mm/h)	10.51 ± 4.80	33.68 ± 4.42	42.45 ± 7.87	^*^0.0054; ^#^<0.001
CPI (units)		52.29 ± 10.78	39.04 ± 12.18	<0.001
Macrophages (%)	78.35 ± 15.38	76.59 ± 12.53	68.32 ± 19.86	^*^0.949; ^#^0.115
Lymphocytes (%)	20.58 ± 8.25	10.58 ± 3.75	32.44 ± 11.32	^*^0.005; ^#^<0.001
Polymorphonuclear (%)	4.38 ± 1.43	8.85 ± 4.38	15.39 ± 8.39	^*^0.012; ^#^<0.001
CRP (mg/dl)	4.79 ± 2.41	11.61 ± 3.14	17.64 ± 4.57	^*^<0.001; ^#^<0.001
IgG (g/L)	11.38 ± 2.59	14.61 ± 4.63	13.50 ± 4.72	^*^0.060; ^#^0.197
IgA (g/L)	2.49 ± 0.88	2.53 ± 0.95	2.26 ± 0.91	^*^0.991; ^#^0.666
IgM (g/L)	2.16 ± 1.14	1.94 ± 0.97	1.84 ± 1.00	^*^0.803; ^#^0.547
DAS28 score	0 ± 0	0 ± 0	5.56 ± 1.76	^*^^,^^#^<0.001
RF positive rate (%)	0 (0/20)	0 (0/16)	75.00 (21/28)	^*^^,^^#^<0.001
Anti-CCP positive rate (%)	0 (0/20)	0 (0/16)	82.14 (23/28)	^*^^,^^#^<0.001

Measurement data were expressed as mean ± SD. Unpaired *t*-test was used for comparison between the two groups. One-way ANOVA was used for comparison among groups. The count data were expressed by ratio, and Fisher’s exact test was used. FEV1, forced expiratory volume in 1 s; FVC, forced vital capacity; DLCO, carbon monoxide diffusion capacity.

* vs. controls.

# vs. IPF patients.

**Figure 1:**
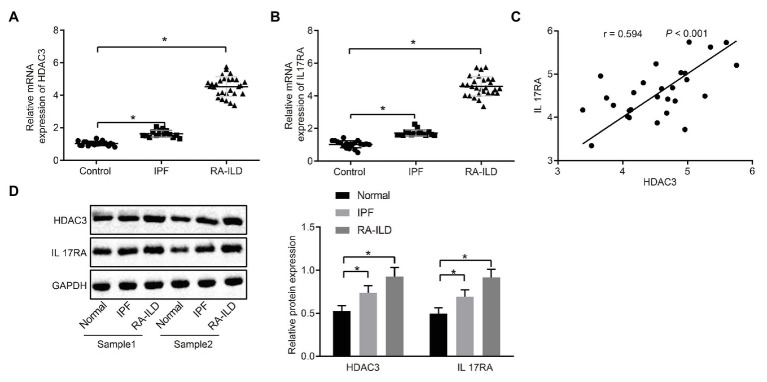
High expression of histone deacetylase 3 (HDAC3) and interleukin 17 receptor A (IL17RA) in patients with idiopathic pulmonary fibrosis (IPF) or rheumatoid arthritis-interstitial lung disease (RA-ILD). **(A)** Reverse transcription quantitative PCR (RT-qPCR) examining HDAC3 expression in lung tissues of controls (*n* = 20), IPF patients (*n* = 16) and RA-ILD patients (*n* = 28). **(B)** RT-qPCR examining IL17RA expression in the lung tissues of controls (*n* = 20), IPF (*n* = 16), and RA-ILD patients (*n* = 28). **(C)** Analysis of the correlation between HDAC3 and IL17RA expression in RA-ILD. **(D)** Western blot examining the protein expression of HDAC3 and IL17RA in lung tissues of controls, IPF and RA-ILD patients. ^*^p < 0.05 compared with controls. Comparisons among multiple groups were analyzed by one-way ANOVA, followed with Tucky’s *post hoc* test. Pearson’s correlation was used to analyze the correlation between HDAC3 and IL17RA expression.

### High Expression of HDAC3 and IL17RA in Lung Tissues of RA-ILD Mice and Lung Fibroblasts

**Figure 2:**
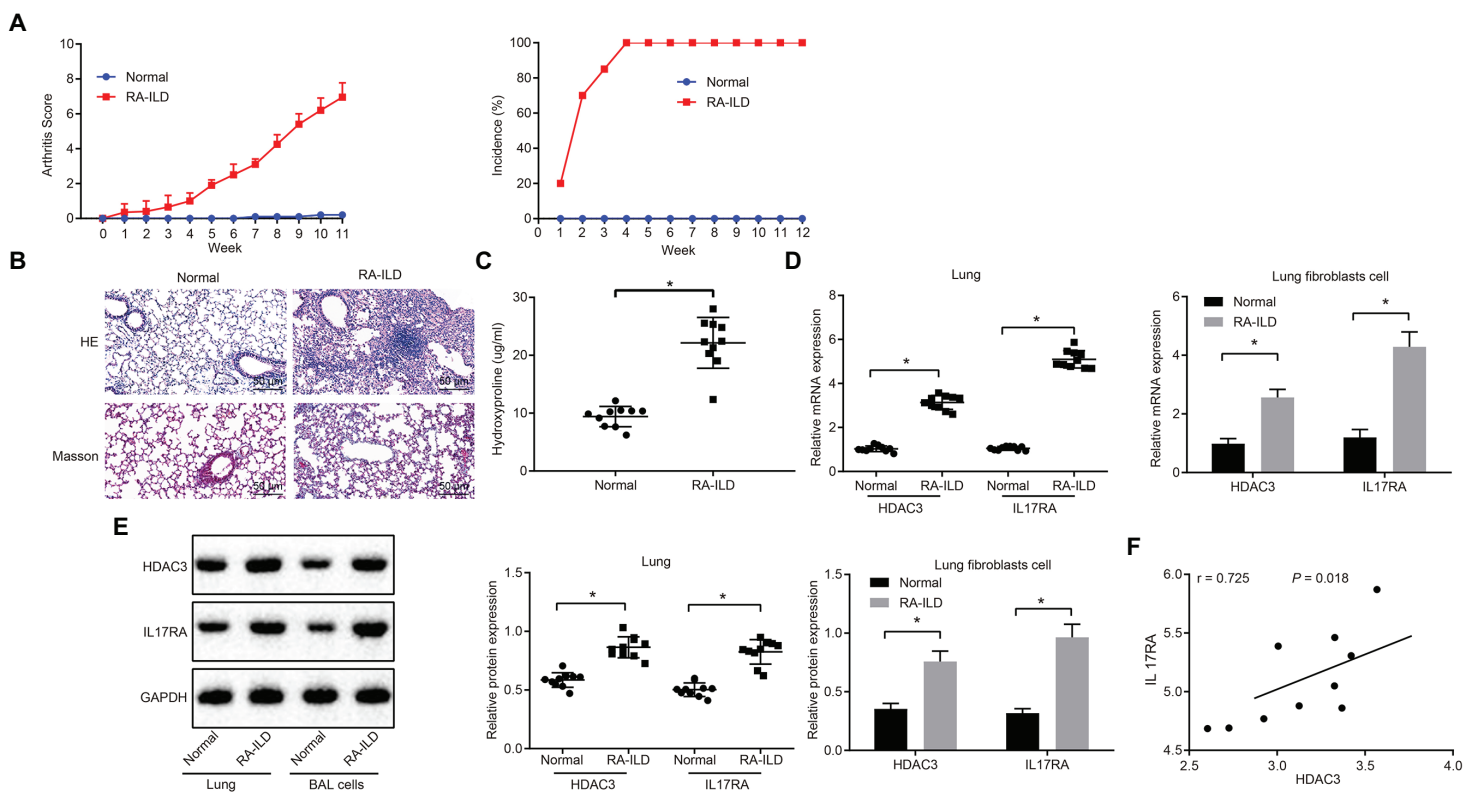
HDAC3 and IL17RA are highly expressed in lung tissues and lung fibroblasts of RA-ILD mice model. **(A)** Arthritis score and morbidity in mice. **(B)** HE (×200, above) and Masson (×200, below) staining of inflammation of lung tissues and pulmonary fibrosis of control mice and RA-ILD mice. **(C)** The hydroxyproline amount in control mice and RA-ILD mice. **(D)** RT-qPCR examining the expression of HDAC3 and IL17RA in mouse lung tissues and lung fibroblasts (*n* = 10). **(E)** Western blot analysis of HDAC3 and IL17RA expression in RA-ILD mice. **(F)** Analysis of the relationship between HDAC3 and IL17RA in RA-ILD mice. ^*^p < 0.05 compared with control mice. Repeated measure ANOVA was used for comparison between two groups at different time points, and independent sample *t*-test was used for comparison between two groups. Pearson correlation analysis of HDAC3 and IL17RA in RA-ILD mice.

Injection of zymosan into SKG mice can induce RA-ILD (Redente et al., 2018; Sendo et al., 2019). SKG female mice were injected with zymosan to construct RA-ILD mouse models. The joint disease and morbidity of the mice were evaluated weekly. It was found that at 10–12 weeks, the mice suffered from severe joint swelling (Figure 2A). Masson and HE staining of the left lung (Figure 2B) showed aggravated pulmonary fibrosis and inflammation around the alveoli of the RA-ILD animal. Moreover, the RA-ILD model mice had higher hydroxyproline than the control animals (Figure 2C). Results of RT-qPCR assay (Figure 2D) revealed that HDAC and IL17RA expression was higher in the lung tissues and lung fibroblasts of mice with RA-ILD than in the controls. The results of immunofluorescence staining showed that HDAC was mainly expressed in the nucleus, while IL-17RA was mainly expressed in the caryoplasm. Besides, the expression of HDAC3, IL17RA, and α-SMA is noticeably higher than the control (Supplementary Figure 1B). Western blot results were consistent with RT-qPCR data (Figure 2E). Correlation analysis (Figure 2F) showed that HDAC3 and IL17RA were also positively correlated in lung tissues of the RA-ILD mice. The above results indicated that HDAC3 and IL17RA were highly expressed in lung tissues and lung fibroblasts of RA-ILD mouse models.

### HDAC3 Inhibited miR-19a-3p Expression in Lung Fibroblasts of RA-ILD Mice to Regulate Pulmonary Fibrosis

**Figure 3:**
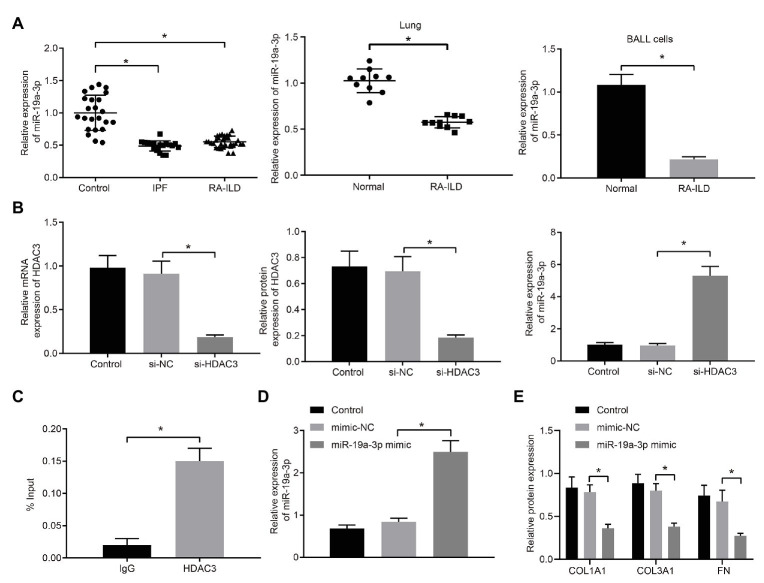
HDAC3 inhibited the expression of microRNA-19a-3p (miR-19a-3p) by deacetylation. **(A)** RT-qPCR examining miR-19a-3p expression in lung tissues of RA-ILD patients as well as lung tissues and lung fibroblasts of RA-ILD mice. **(B)** Silencing efficiency of HDAC3 and expression of miR-19a-3p after silencing HDAC3. **(C)** Chromatin immunoprecipitation (ChIP) examining the enrichment of immunoglobin G (IgG) and HDAC3 in the miR-19a-3p promoter region, respectively. **(D)** The efficiency of miR-19a-3p over-expression. **(E)** Expression of COL1A1, COL3A1, and FN protein after over-expressing miR-19a-3p. ^*^p < 0.05 compared with the corresponding controls. An independent sample *t*-test was used for comparison between the two groups, and one-way ANOVA was used for comparison among groups.

Expression of miR-19a-3p was examined in RA-ILD patients, mouse lung tissues, and mouse lung fibroblasts. The results (Figure 3A) showed that miR-19a-3p was poorly expressed in lung tissues of RA-ILD patients and mice.

The regulatory mechanism of HDAC3 and miR-19a-3p in RA-ILD was further investigated. HDAC3 in lung fibroblasts of primary mice with RA-ILD was silenced by transfection of siRNA. The results (Figure 3B) demonstrated that HDAC3 mRNA and protein expression decreased in the cells transfected with si-HDAC3, while the expression of miR-19a-3p increased. The ChIP assay (Figure 3C) examined the enrichment of HDAC3 in the miR-19a-3p promoter region and found that HDAC3 was enriched in the miR-19a-3p promoter region. These results supported that HDAC3 could recruit miR-19a-3p region to inhibit miR-19a-3p expression.

To further investigate if miR-19a-3p involved in regulation of pulmonary fibrosis in RA-ILD, miR-19a-3p mimic was transfected into lung fibroblasts from RA-ILD mice. RT-qPCR (Figure 3D) confirmed the upregulation of miR-19a-3p by miR-19a-3p mimic. Additionally, over-expression of miR-19a-3p inhibited COL1A1, COL3A1, and FN protein expression in mouse lung fibroblasts (Figure 3E). The results indicated that HDAC3 was elevated while miR-19a-3p was reduced in the RA-ILD mice and RA-ILD patients, and miR-19a-3p enhanced the expression of fibroblast marker gene in mouse lung fibroblasts.

### IL17RA Was a Downstream Target Gene of miR-19a-3p

**Figure 4:**
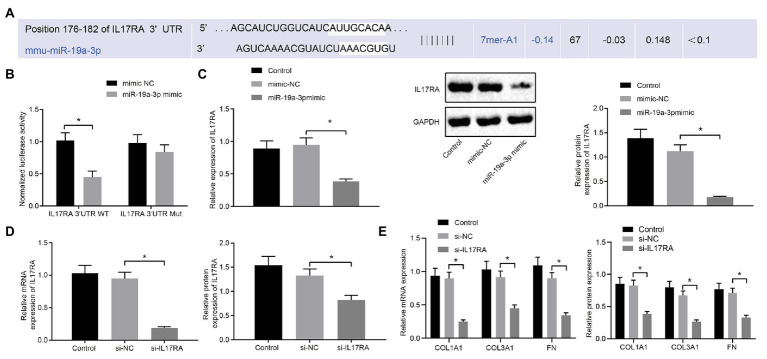
IL17RA was the target gene of miR-19a-3p. **(A)** TargetScan predicting the downstream target gene of miR-19a-3p. **(B)** Dual luciferase reporter assay examining luciferase activity in different groups. **(C)** RT-qPCR and western blot examining IL17RA expression with over-expressing miR-19a-3p. **(D)** IL17RA inhibition efficiency detected by RT-qPCR and Western blot analysis. **(E)** The mRNA and protein expression of COL1A1, COL3A1, and FN after inhibiting IL17RA detected by RT-qPCR and Western blot analysis. ^*^p < 0.05 compared with the corresponding controls. Independent sample *t*-test was used for comparison between the two groups, and one-way ANOVA was used for comparison among groups.

The binding site of miR-19a-3p and IL17RA in mouse cells was predicted by TargetScan website prediction (Figure 4A). To investigate whether IL17RA is a target gene for miR-19a-3p in lung fibroblasts of RA-ILD mice, the binding of miR-19a-3p and IL17RA 3'UTR was examined by dual luciferase reporter assay in HEK293T cells. The results (Figure 4B) indicated that overexpression of miR-19-3p inhibited the 3'UTR WT of IL17RA, but had no significant effect on the luciferase activity of the 3'UTR MUT of IL17RA. RT-qPCR and Western blot analysis found that (Figure 4C) IL17RA expression was decreased after over-expressing miR-19a-3p.

Additionally, RT-qPCR and Western blot analysis (Figure 4D) confirmed the decreased mRNA and protein levels of IL17RA in the lung fibroblasts transfected with si-IL17RA. Moreover, expression of COL1A1, COL3A1 and FN in the lung fibroblasts transfected with si-IL17RA also decreased (Figure 4E). Thus, IL17RA was a target gene of miR-19a-3p in lung fibroblasts of RA-ILD mice, and miR-19a-3p could downregulate IL17RA to inhibit the expression of COL1A1, COL3A1, and FN.

### HDAC3 Promoted RA-ILD Fibrosis Through miR-19a-3p-Dependent IL17RA

**Figure 5:**
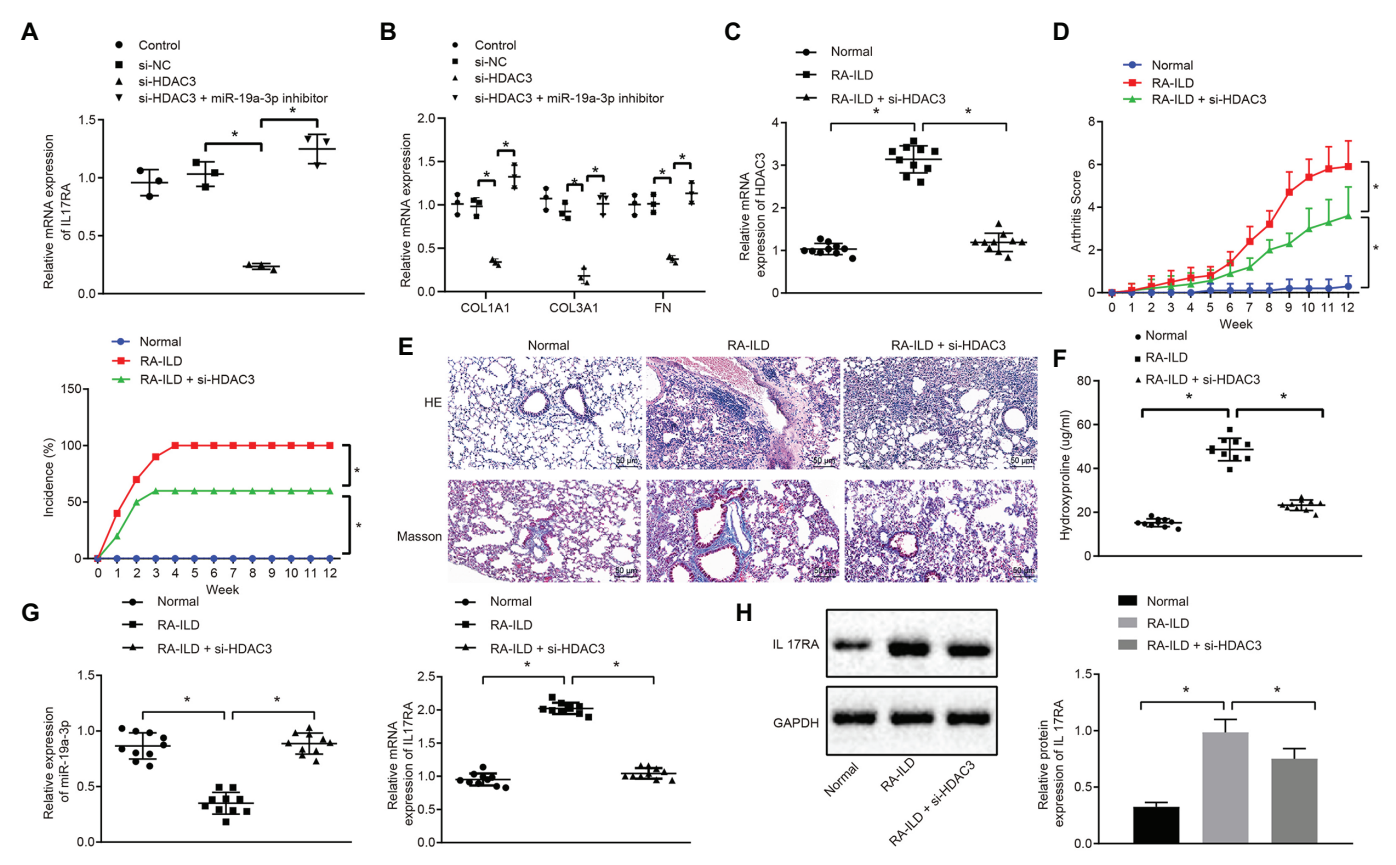
The process of interstitial pulmonary fibrosis in RA mice was promoted by HDAC3 through miR-19a-3p targeting IL17RA. **(A)** RT-qPCR results of IL17RA expression after silencing HDAC3 or miR-19-3p. **(B)** RT-qPCR results of the expression of COL1A1, COL3A1, and FN after silencing HDAC3 or miR-19-3p. **(C)** HDAC3 mRNA expression in lung tissues of mice in each group. **(D)** Examination of arthritis scores and morbidity in different mice. **(E)** HE (×200, above) and Masson (×200, below) staining examining the degree of damage in lung tissues of mice in each group. **(F)** Examination of the change of hydroxyproline amount in the lung tissues of mice in each group. **(G)** Examination of the expression of miR-19a-3p in lung tissues of mice in each group. **(H)** Examination of the expression of IL17RA in lung tissues of mice in each group. ^*^p < 0.05 compared with control. Multiple comparisons at different time points were analyzed by repeated measures ANOVA, and comparisons among groups were applied using one-way ANOVA.

To further explore whether HDAC3 induces the occurrence of RA-ILD through regulating miR-19a-3p to elevate IL17RA protein expression, the expression of IL17RA was examined in cells of silencing HDAC3 or miR-19a-3p. The results (Figure 5A) displayed that silencing HDAC3 alone inhibited IL17RA expression in mouse lung fibroblasts, whilst silencing HDAC3 and miR-19a-3p together up-regulated IL17RA expression in mouse lung fibroblasts. Besides, silencing HDAC3 decreased the expression of COL1A1, COL3A1, and FN in mouse lung fibroblasts, while those expressions were increased after downregulation of both HDAC3 and miR-19-3p (Figure 5B). Arthritis and pulmonary fibrosis were studied in RA-ILD mouse model. Furthermore, the lung fibroblasts were transfected with adenovirus-mediated si-HDAC3. The expression determination results (Figure 5C) noted that HDAC3 was highly expressed in lung tissues of RA-ILD mice than in lung tissues of RA-ILD mice transfected with si-HDAC3. Then, we found that (Figure 5D) silencing HDAC3 reduced arthritic scores and morbidity in RA-ILD mice. HE and Masson staining results showed that silencing HDAC3 improved the condition of inflammation around the alveoli in mice, and pulmonary fibrosis was alleviated after silencing HDAC3 (Figure 5E). Besides, the hydroxyproline expression in the lung tissue of RA-ILD mice decreased after silencing HDAC3 (Figure 5F). Furthermore, RT-qPCR and Western blot analysis results showed that miR-19a-3p expression was elevated but IL17RA expression was noticeably decreased in lung tissues of RA-ILD mice after silencing HDAC3 (Figures 5G,H). These results demonstrated that HDAC3 regulated the process of RA-ILD in mice through miR-19a-3p-dependent IL17RA.

## Discussion

In RA, the pattern of ILD is the most common interstitial pneumonia (Wells and Denton, 2014). Almost 10% of patients with RA have displayed clinically evident ILD (Paulin et al., 2015). The chronic and progressive fibrosis of lung is the feature of chronic ILD, which includes IPF (Kim et al., 2018). Patients with ILD fibrosis may develop a progressive phenotype featured by self-sustaining fibrosis, lung function declining, to lower life quality and even early death (Cottin et al., 2019). RA patients with ILD have three times higher mortality than those without ILD (Paulin et al., 2017). RA-ILD patients usually have high morbidity and mortality (Morisset et al., 2017). Recent study shows that immunosuppressive treatment is an effective way to relieve exacerbations of RA-ILD (Ota et al., 2017). In this study, we investigated the role of miR-19-3p in RA-ILD fibrosis pathogenesis. We have shown evidence that HDAC3 can negatively regulate miR-19a-3p to increase IL17RA expression, thereby promoting the progression of RA-ILD fibrosis.

It has been documented that HDACs are an important family of 18 isozymes, which are being classified as targets for many diseases (Stoddard et al., 2019). Our study revealed that HDAC3 and IL17RA were highly expressed in lung tissues of RA-ILD patients and in the lung fibroblasts of mouse model. Similarly, inhibiting HDAC3 in RA fibroblast-like synoviocytes exhibits the effects of pan-HDACi (a member of the second generation HDAC inhibitors) in inhibiting the expression of inflammatory genes (Angiolilli et al., 2017; Simic and Sang, 2019). Mounting evidence shows that aberrant histone acetylation and HDAC are increasingly considered to play critical roles in the onset of RA (Li et al., 2018).

In this study, the results revealed that miR-19a-3p was poorly expressed in the mouse model of RA-ILD, and over-expression of miR-19a-3p inhibited the expression of COL1A1, COL3A1, and FN. Consistent with our findings, previous study has reported that the miR-19a-3p was low-expressed in the patients with RA (Chen et al., 2019). The level of circulating miR-19a-3p was lower in the patients with cardiovascular disease compared with controls (Marques et al., 2017). Moreover, the low expression of miR-19a-3p and miR-19b-3p in Crohn’s disease with intestinal fibrosis regulates the development of Crohn’s disease fibrosis and subsequent stenosis formation (Lewis et al., 2015). It is known that COL1A1, COL3A1, and FN were considered as marker genes of fibrosis. For example, the expression of COL1A1, FN, and α-SMA increased in one fibrotic muscle model (Jiang et al., 2018). Previous research has proposed that PolyG decreased the expression of fibrosis-related transcription factors, COL1A1 and COL3A1, in the lungs of rats exposed to silica (Yang et al., 2019). COL3A1 is a critical part of normal collagen I fibrillogenesis in many organs (Wang et al., 2015). In addition, the pulmonary indexes, pulmonary fibrosis, FN, and Collagen I, all increased in pulmonary tissues (Liao et al., 2017). In this study, we provided evidence proving that overexpression of miR-19a-3p could improve the ILD.

We further provided evidence that HDAC3 inhibited the expression of miR-19a-3p to regulate pulmonary fibrosis. Consistently, HDAC3 inhibits the expression of miR-17-9 to release proper TGFβ signaling in lung sacculation (Wang et al., 2016). The over-expression of HDAC3 mediated by lentivirus inhibited the expression of miR-10a (Shan et al., 2016). HDAC3 plays important roles in miR-30d repression and podocyte injuries induced by TGFβ (Liu et al., 2016). Besides, we identified IL17RA as a target gene of miR-19a-3p. HDAC3 promoted RA-ILD fibrosis *via* regulating miR-19a-dependent expression of IL17RA, and IL17RA facilitated the progression of RA fibrosis. Consistent with our findings, IL17 has been considered to be associated with organ fibrosis (Ramani et al., 2018). The lack of IL17RA changes the morphology of homeostatic tissue macrophage and inhibits renal inflammation and fibrosis (Ge et al., 2014). The IL17A/IL17RA axis plays important role in the fibrosis of murine airway (Yanagisawa et al., 2017). As to those marker genes of fibrosis, we found that IL17RA could increase the expressions of COL1A1, COL3A1, and FN proteins. Consistently, IL17C-treated Apoe^(−/−)^ aortas increased the expression of COL1A1 *in vitro* (Butcher et al., 2016). IL17 stimulation induced multiple cellular responses, such as the significant increase in mRNA expression of COL1A1 (Lin et al., 2015). In this research, miR-19a-3p decreased the expression of fibrosis marker genes COL1A1, COL3A1, and FN through inhibiting IL17RA expression.

## Conclusion

**Figure 6:**
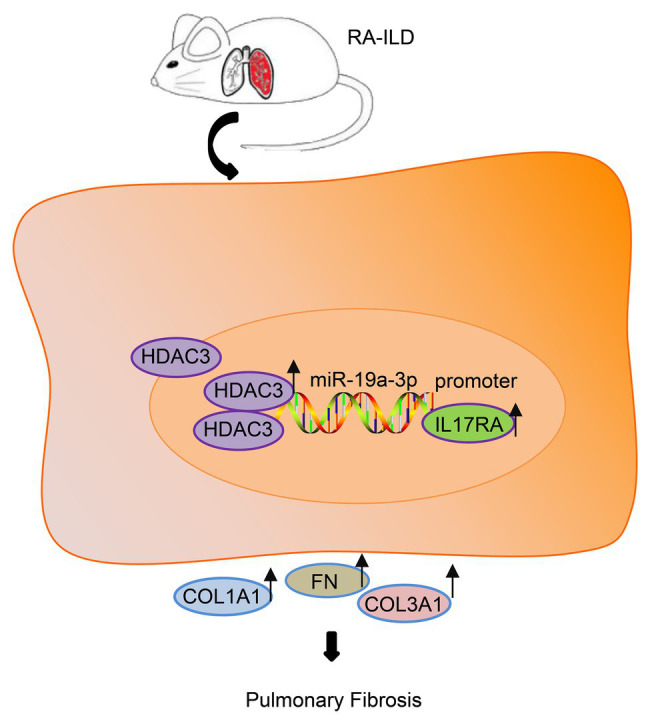
The histone deacetylase HDAC3 promoted the development of RA-ILD fibrosis through increasing miR-19a-3p-dependent IL17RA expression.

On the basis of the above findings, HDAC3 and IL17RA were over-expressed in patients and mouse model with RA while miR-19a-3p was under-expressed. IL17RA is the target gene of miR-19a-3p, and miR-19a-3p could negatively regulate IL17RA to inhibit RA-ILD fibrosis (Figure 6). In summary, HDAC3 facilitated the development of RA-ILD fibrosis through upregulating miR-19a-3p-mediated IL17RA expression. However, the diagnosis of RA-ILD is based on the RA classification standard in 1987, so the differences between the 1987 criteria and the 2010 criteria may affect the credibility of the study conclusions.

## Data Availability Statement

All datasets presented in this study are included in the article/Supplementary Material.

## Ethics Statement

The studies involving human participants were reviewed and approved by the Ethics Committee of Affiliated Hospital of Shaanxi University of Chinese Medicine. The patients/participants provided their written informed consent to participate in this study. The animal study was reviewed and approved by Animal Ethics Committee of Shaanxi University of Chinese Medicine.

## Author Contributions

HY, LJ, and NY designed the study. LJ, HD, YY, and YB collated the data, carried out data analyses, and produced the initial draft of the manuscript. HY and YB contributed to drafting the manuscript. All authors have read and approved the final submitted manuscript.

### Conflict of Interest

The authors declare that the research was conducted in the absence of any commercial or financial relationships that could be construed as a potential conflict of interest.

## Supplementary Material

Supplementary Figure 1 **(A)** Immunohistochemistry localizing the expression of HDAC3 and IL17RA in lung tissues of controls, IPF and RA-ILD patients (×400). **(B)** Cell immunofluorescence detection of the co-location of α-SMA with HDAC3 and IL17RA in lung fibroblasts (×200).
