# Supplementary figures and images for: Histone Deacetylase 3-Mediated Inhibition of microRNA-19a-3p Facilitates the Development of Rheumatoid Arthritis-Associated Interstitial Lung Disease
Source: Front Physiol. 2020 Dec 4;11:549656. doi: 10.3389/fphys.2020.549656 (PMC7746846; doi:10.3389/fphys.2020.549656)

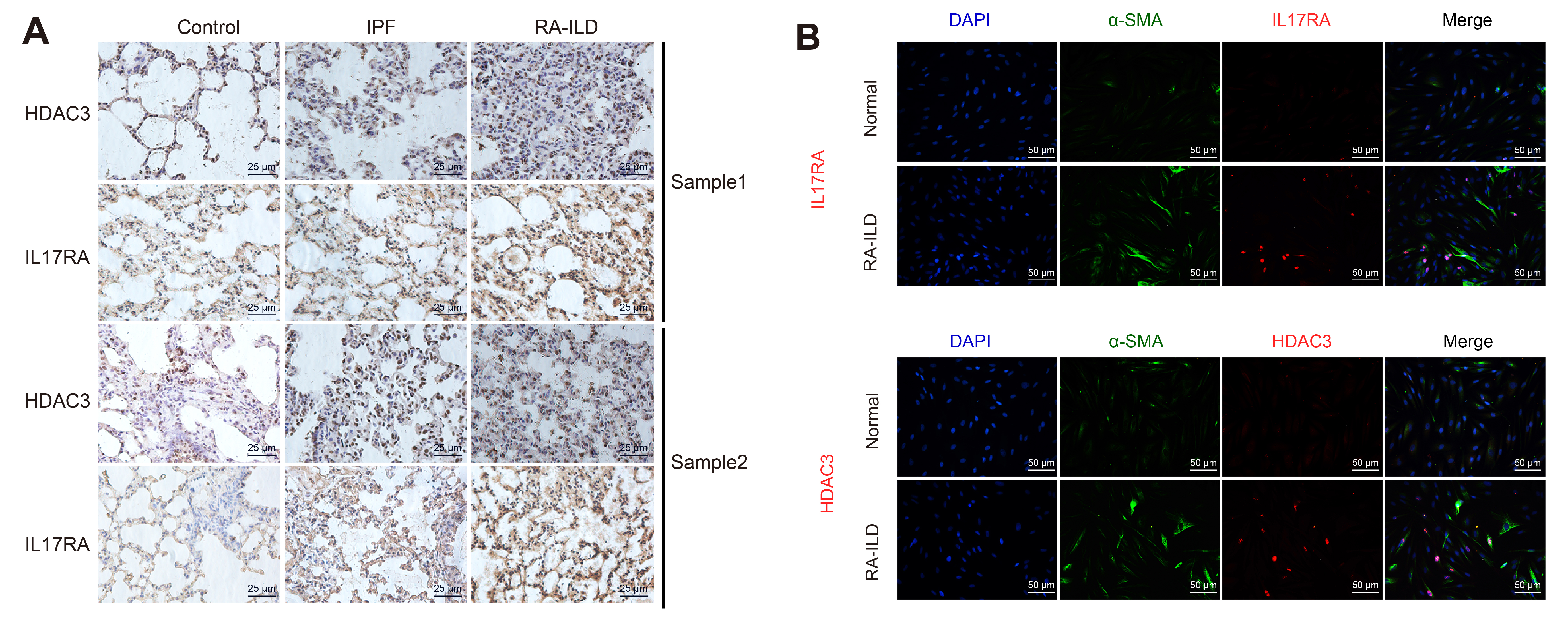

Supplement: Supplementary Figure 1 — (A) Immunohistochemistry localizing the expression of HDAC3 and IL17RA in lung tissues of controls, IPF and RA-ILD patients (×400). (B) Cell immunofluorescence detection of the co-location of α-SMA with HDAC3 and IL17RA in lung fibroblasts (×200). [file Image_1.jpeg]
